# Effects of Traumeel (Tr14) on Exercise-Induced Muscle Damage Response in Healthy Subjects: A Double-Blind RCT

**DOI:** 10.1155/2016/1693918

**Published:** 2016-07-05

**Authors:** Kerstin Muders, Christian Pilat, Vanessa Deuster, Torsten Frech, Karsten Krüger, Jörn Pons-Kühnemann, Frank-Christoph Mooren

**Affiliations:** ^1^Department of Sports Medicine, Justus Liebig University, Kugelberg 62, 35394 Giessen, Germany; ^2^Medical Statistics, Justus Liebig University, Heinrich-Buff-Ring 44, 35392 Giessen, Germany

## Abstract

The present double-blind, randomized, placebo-controlled clinical trial intended to test whether ingestion of a natural combination medicine (Tr14 tablets) affects serum muscle damage and inflammatory immune response after downhill running. 96 male subjects received Tr14 tablets, which consist of 14 diluted biological and mineral components, or a placebo for 72 h after the exercise test, respectively. Changes in postexercise levels of various serum muscle damage and immunological markers were investigated. The area under the curve with respect to the increase (AUC_i_) of perceived pain score and creatine kinase (CK) were defined as primary outcome measures. While for CK the *p* value of the difference between the two groups is borderline, the pain score and muscle strength were not statistically significant. However, a trend towards lower levels of muscle damage (CK, *p* = 0.05; LDH, *p* = 0.06) in the Tr14 group was shown. Less pronounced lymphopenia (*p* = 0.02), a trend towards a lower expression of CD69 count (*p* = 0.07), and antigen-stimulated ICAM-1 (*p* = 0.01) were found in the verum group. The Tr14 group showed a tendentially lower increase of neutrophils (*p* = 0.10), BDNF (*p* = 0.03), stem cell factor (*p* = 0.09), and GM-CSF (*p* = 0.09) to higher levels. The results of the current study indicate that Tr14 seems to limit exercise-induced muscle damage most likely via attenuation of both innate and adaptive immune responses. This study was registered with ClinicalTrials.gov (NCT01912469).

## 1. Introduction

An acute bout of physical exercise depending on duration and intensity is known to induce changes of the immune response of both the innate and the adaptive immune system. Thereby, the numbers and functions of circulating leukocytes are affected. Changes of leukocytes are regularly accompanied by an exercise-induced increase of inflammatory cytokines such as IL-6, IL-10, and IL-1ra [1]. Eccentric exercise like downhill running is a special exercise mode which is known to cause substantial muscle damage followed by a pronounced inflammatory response. This muscle damage is accompanied by muscle soreness, which regularly occurs 12–48 hours after the eccentric exercise bout and which was termed delayed onset of muscle soreness (DOMS). DOMS is furthermore accompanied by stiffness or tenderness on palpation and a loss of muscle force [2, 3].

Actually, there are two different mechanisms that explain the induction of DOMS. Firstly, mechanical stress generated during the eccentric exercise is considered as one mechanism [4]. Thereby, the increased tension per individual cross bridge and the stretching forces on sarcomeres induce microtrauma in muscle fibers [3]. Microscopically, observations show ruptures of Z-discs and A-bands and the subsequent dissolution of sarcomere structures in individual muscle fibers [5, 6]. Structural damage is accompanied by increased cytosolic calcium concentrations, which activate proteolytic enzymes and increase cell membrane permeability [3]. The result is release of muscle enzymes such as creatine kinase (CK) into the interstitial fluid [7]. Secondly, it is suggested that DOMS is based on inflammatory responses after eccentric exercise [4]. Thereby, several studies described the occurrence of a local inflammation in muscle tissue. Within this inflammation, migration of neutrophils and macrophages into the damaged tissue several hours after exercise is observed. Neutrophils eliminate cell fragments and perform reorganization processes via reactive oxygen species. Both neutrophils and macrophages are involved in the release of cytokines such as IL-1, TNF-*α*, and IL-6 [3, 8].

Several approaches were done to prevent muscle soreness and stimulate regeneration. Some studies investigated the effect of nonsteroidal anti-inflammatory drugs (NSAIDs) or antioxidants [9–11]. Others examined the application of various physical therapy methods [12, 13]. Regarding the use of antioxidants (e.g., vitamin C and vitamin E), no clear effect on reduction of muscle damage was found [11]. Similarly, neither cryotherapy nor stretching exercise was effective in treating exercise-induced muscle damage [12, 13]. In contrast, studies about the administration of NSAIDs have shown contradictory results. While some studies showed no changes after treatment with NSAIDs [9, 10], others demonstrated lower symptoms of DOMS [14, 15]. However, athletes have to consider several side effects of the regular use of NSAIDs such as upper gastrointestinal bleeding and perforation, renal injury, liver injury, and heart failure [16].

Therefore, the current study intended to test the effect of the well tolerated natural combination medicine Tr14 on DOMS. Tr14 consists of 14 diluted biological and mineral components. It is composed mainly of plants of the Compositae family (chamomile, common daisy, common marigold, common yarrow, mountain arnica, narrow-leaved purple coneflower, and purple coneflower). They showed in various studies immunomodulating effects. For example, for both chamomile and coneflower, anti-inflammatory effects have been shown [17, 18]. The residual plant components of Tr14 are composed of different plant families (comfrey, common witch-hazel, deadly nightshade, St. John's wort, and wolf's bane). For St. John's wort, anti-inflammatory effects have been shown in an in vivo study [18]. Tr14 also consists of two mineral components (calcium sulfide and Mercurio-amidonitrate). Evidence for immunomodulatory effects of Tr14 comes from humans [19–21], animals [22, 23], and in vitro [21, 24] studies. Clinical evidence for Tr14 and composition in more detail was reviewed by Schneider [25]. González de Vega et al. demonstrated in a human clinical trial that Tr14 decreased pain and improved joint function in acute ankle sprain when topically applied [19]. Recently, we observed in a human clinical trial that Tr14 has effects on various immune parameters of the exercise-induced immune response [20]. Here, we showed that Tr14 reduced the inflammatory response of the innate immune system while the response of proinflammatory cytokines was promoted. The animal study of Oberbaum et al. demonstrated that Tr14 increased IL-1*β* in an in vivo sepsis model in rats [23]. Furthermore, Lussignoli et al. showed that Tr14 significantly reduced IL-6 production in rats with edema [22]. In contrast, Porozov et al. showed in an “in vitro” study that Tr14 inhibits the secretion of proinflammatory cytokines such as IL-1*β*, CXCL8, and TNF-*α* in resting as well as activated immune cells [24].

The present double-blind, randomized, placebo-controlled study follows up our research on effects of Tr14 in strenuous exercise paradigm, this time examining exercise-induced muscle soreness. We hypothesized that Tr14 is effective in limiting exercise-induced muscle damage and inflammatory response. The results might be helpful in optimizing load-recovery cycles during various training regimes thereby improving the training effectivity.

## 2. Methods

### 2.1. Study Design

**Figure 1:**
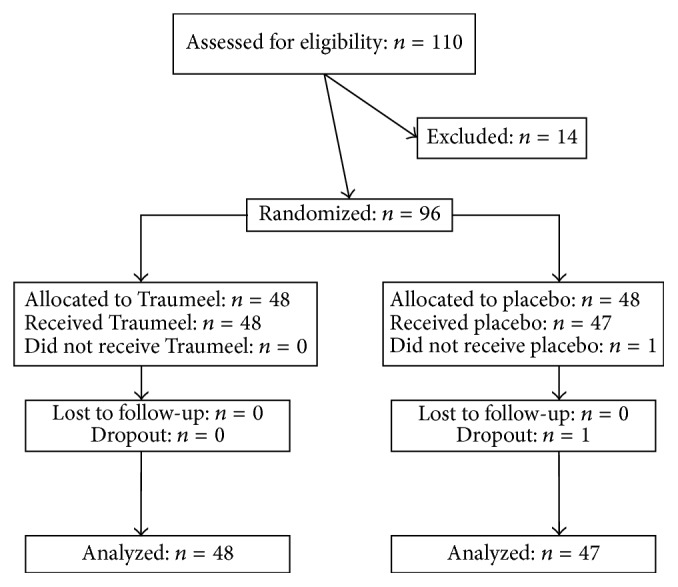
Study flow chart. 110 subjects were assessed for eligibility in the clinical trial and thereof 96 subjects were randomized. There was one dropout in the placebo group.

**Figure 2:**
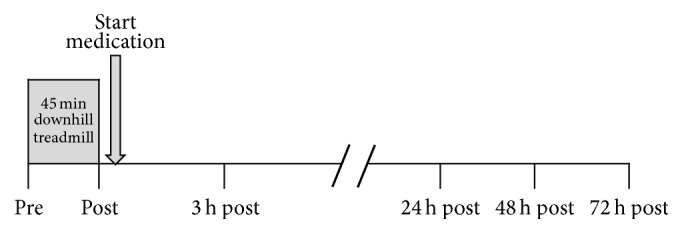
Experimental design. The figure shows the different time points of data collection. On day one, the data collection was performed immediately before, immediately after, and three hours after exercise (45 min downhill running). Follow-up visits were performed 24 h, 48 h, and 72 h after exercise. The intake of the study medication started directly after exercise.

The present study was conducted as a double-blind, randomized, placebo-controlled clinical trial. This monocenter study was performed at the Department of Sports Medicine, University of Giessen. The study protocol was approved by the local ethics committee and the Federal Institute for Drugs and Medical Devices (EudraCT 2009-010898-21). The study was registered with ClinicalTrials.gov (NCT01912469). The study flow chart according to CONSORT is shown in Figure 1. Initially, 110 subjects were examined for their medically approved unrestricted participation in sport using an incremental protocol on a treadmill. After the test, 14 subjects were not included into the study because they did not meet the inclusion criteria of the study (see below). Hence, a total of 96 subjects were enrolled in the study and randomized either to Tr14 (*n* = 48) or to placebo (*n* = 48). A total of 95 subjects were analyzed because of one dropout due to a study related adverse event in the placebo group before intake of study medication. The duration of subject study participation was four days. The screening was performed within 8 days prior to study entry. On day one, the subjects came fasting to the study center and got a standardized breakfast and the following procedure was performed: inclusion/exclusion criteria rechecking, subject randomization, acute bout of eccentric exercise, data collection at three different time points, and dispensing of study medication (Figure 2). Before, immediately after, and three hours after exercise, various analyses were performed (blood samples, strength measurement, and SF-McGill Pain Questionnaire). The subjects were not allowed to consume any food until three hours after exercise. In contrast, water was allowed ad libitum. Follow-up visits were performed at 24 h, 48 h, and 72 h after exercise. The subjects were requested 48 h prior to study participation and during the study to refrain from strenuous physical activity.

### 2.2. Subjects

The subject characteristics showed only marginal differences between the two groups (Table 1). Subjects were included if they met all inclusion criteria and none of the exclusion criteria. Subjects were eligible for the study if they were male, were aged ≥ 18 and ≤40 years, had BMI ≥ 18.5 and < 27.5 kg/m^2^, had maximum relative oxygen uptake (VO_2max_) < 53 mL/kg·min, were healthy, were nonsmokers, had medically approved unrestricted sports participation as shown by diagnostic performance test conducted on treadmill for no longer than three months prior to study entry, and were willing to provide informed consent. If any of the following exclusion criteria existed, the subjects were not included in the study: regular eccentric exercise training, weekly training volume ≥ 6 hours, use of dietary supplements, chronic immune deficiency, current infection, heart and/or circulation disorders, musculoskeletal disorders, nothing that requires a systemic therapy, hypersensitivity to botanicals of the Compositae family, lactose intolerance, illicit drug or alcohol abuse, and participation in another clinical trial within four weeks prior to study entry.

### 2.3. Exercise Protocol

The subjects were instructed to run downhill (10% incline) on a treadmill (h/p/cosmos quasar med 4.0, h/p/cosmos Sports & Medical GmbH, Nussdorf, Traunstein, Germany) as a means to eccentric exercise at a heart rate (HR) corresponding to 80% of VO_2max_. The duration of the eccentric exercise was set to 45 min unless the subjects were forced to quit earlier due to exhaustion. The protocol began with a short warm-up. Afterwards, the treadmill speed increased to 8 km/h and the downhill running started. The heart rate was regulated manually by speed adjustment on the running belt.

### 2.4. Study Medication

Tr14 tablets are a registered natural combination medication. The study medication was manufactured by Biologische Heilmittel Heel GmbH, Baden-Baden, according to the German Homeopathic Pharmacopoeia (HAB), current EU-GMP guidelines, and pertinent national regulations. Participants and investigators were blinded to the study medication by identical color, smell, taste, and size. The 1-to-1 randomization with a block size of eight was conducted by DATAMAP GmbH, Freiburg, Germany, according to the company internal SOP. The subjects were randomized on day one directly before the first study related procedure was carried out. Time of randomization and study entry were defined by taking the first blood sample. Tr14 consists of the following active components (D stands for dilutions in increments, amounts per 300 mg):* Achillea millefolium* (D3, 15 mg),* Aconitum napellus* (D3, 30 mg),* Arnica montana* (D2, 15 mg),* Atropa belladonna* (D4, 75 mg),* Bellis perennis* (D2, 6 mg),* Calendula officinalis* (D2, 15 mg),* Chamomilla recutita* (D3, 24 mg),* Echinacea angustifolia* (D2, 6 mg),* Echinacea purpurea* (D2, 6 mg),* Hamamelis virginiana* (D2, 15 mg),* Hepar sulfuris* (D8, 30 mg),* Hypericum perforatum* (D2, 3 mg),* Mercurius solubilis Hahnemanni* (D8, 30 mg), and* Symphytum officinale* (D8, 24 mg); and it also consists of the following inactive components: lactose (6 mg) and magnesium stearate (1.5 mg). All components are prepared according to Homeopathic Pharmacopoeia. The placebo tablet consists of 300 mg lactose monohydrate and 1.5 mg magnesium stearate.

The subjects received one tablet every 15 minutes until eight tablets were taken (dissolved in the mouth) after the exercise test. In addition, the subjects ingested two tablets 6 h and 10 h after the exercise test. On the following two days, they received two tablets three times a day and on the fourth day they took two tablets in the morning. The total study dose was 26 tablets.

### 2.5. Blood Collection

At each data collection point, approximately 19 mL of blood was taken from the antecubital vein using Safety-Multifly Needle (Sarstedt, Nümbrecht, Germany).

### 2.6. White Blood Cell Count and Lymphocyte Activation and Apoptosis

The tube with EDTA blood (2.7 mL EDTA K tubes, Sarstedt, Nümbrecht, Germany) was analyzed for white blood cell count using the optoelectronic principle (XE2100, Sysmex, Norderstedt, Germany). For the determination of apoptosis and surface markers, the peripheral blood mononuclear cells (PBMCs) were isolated by using lithium-heparinized blood and density gradient centrifugation. Subsequently, the cells were labeled with FITC-conjugated (Annexin V, anti-human CD62L, anti-human CD69, and anti-human CD95R; ImmunoTools, Friesoythe, Germany) and PE-conjugated (anti-human CD95L; BioLegend, San Diego, USA) antibodies. Next, the lymphocytes were gated by flow cytometry and measured (Coulter Epics XL-MCL, Beckman-Coulter, Brea, USA). For more details, see works of our own group [26].

### 2.7. Soluble Inflammatory Mediators

The following parameters were measured using multiplex ELISA (Myriad RBM, Austin, Texas) in stimulated whole-blood cultures: BDNF, CCL2, CCL3, CCL4, CXCL8, CCL11, Factor VII, GM-CSF, ICAM-1, IFN-*γ*, IL-2, IL-3, IL-4, IL-5, IL-6, IL-7, IL-10, IL-12p40, IL-12p70, IL-15, IL-17, IL-18, IL-23, IL-1ra, IL-1*α*, IL-1*β*, MMP-3, MMP-9, SCF, TNF-*α*, TNF-*β*, and VEGF. Here, 1 mL of whole blood was withdrawn in blood collection tubes (TruCulture, EDI GmbH, Reutlingen, Germany) by Safety-Multifly Needle (Sarstedt, Nümbrecht, Germany). The collection tubes contained the stimulants lipopolysaccharide (LPS) from* Escherichia coli* serotype O55:B5 and* Staphylococcus enterotoxin* B (SE-B) in a concentration of 0.1 *µ*g/mL. Each tube was incubated for 24 h in a dry block incubator (VLM GmbH, Bielefeld, Germany) at 37°C. After 24 h, a valve separator was inserted into the tube and the samples were stored at −80°C until further analyses. For more details, see work of our own group [20]. Additionally, six inflammatory mediators were measured in serum samples using multiplex ELISA: CCL2, CXCL8, IL-1ra, IL1- *β*, IL-6, and TNF-*α*.

### 2.8. Muscle Damage and Recovery Markers

The maximum isometric strength of the anterior (extension) and posterior (flexion) thigh muscles was used as a marker of exercise-induced muscle damage and recovery. The Schnell m3-Diagnos multifunctional training and analysis station (Schnell GmbH, Peutenhausen, Germany) was utilized for the measuring of the muscle strength. Furthermore, creatine kinase (CK) and lactate dehydrogenase (LDH) in serum (1.2 mL serum Z tubes, Sarstedt, Nümbrecht, Germany) were additionally used as markers for exercise-induced muscle damage. The Short Form McGill Pain Questionnaire (SF-MPQ) was used to evaluate the experience of pain from the subjects. The SF-MPQ contains ten questions about relating to the sensory level of pain experience and four questions about relating to the affective status. In addition, the SF-MPQ contains a 5-value Likert scale for the overall pain experience and a visual analogue scale (VAS) for the momentary pain intensity [27]. The total score as well as the VAS was evaluated.

### 2.9. Statistical Analysis

Statistical analyses were conducted on the intention-to-treat (ITT) principle and were performed using SAS V 9.3 (SAS Institute, Cary, NC). All randomized and treated subjects were analyzed according to the intention-to-treat principle. The “area under the curve with respect to increase” (AUC_i_) was calculated with reference to time point before exercise. Perceived pain score and CK were defined as the primary outcome measure. AUC_i_ was calculated using the trapezoidal rule [28]. In order to identify a potential difference between the two groups, a median test of AUC_i_ was performed. All other outcome measures were included as secondary outcome measures.

A *p* value of ≤0.05 was accepted as statistically significant. The *p* values of all secondary outcome measures were set to ≤0.05. The *p* values were two-tailed. There was no multiple test adjustment of *α*-error.

## 3. Results

### 3.1. Compliance to Exercise Protocol

Compliance with exercise protocol was assessed using the following variables: completion of exercise protocol over 45 min (Tr14: *n* = 48, 100%; placebo: *n* = 44, 93.6%), time to exhaustion if the subject did not accomplish the exercise test (Tr14: 45 ± 0 min; placebo: 40.3 ± 4.6 min), and average HR throughout the exercise protocol (Tr14: 164 ± 12.9 BPM; placebo: 161.8 ± 10.4 BPM).

### 3.2. Muscle Damage Markers and Recovery

The maximum isometric strength (flexion and extension) decreased after exercise in both groups (Supplemental Table 1 in Supplementary Material available online at http://dx.doi.org/10.1155/2016/1693918). The muscle strength (flexion) showed a minimum immediately after exercise in the Tr14 group and 24 h after exercise in the placebo group. Both groups demonstrated a slight increase 48 h after exercise. In contrast, the muscle strength (extension) indicated a minimum immediately after exercise in the Tr14 group and 3 h after exercise in the placebo group and a slight increase 24 h after exercise in both groups. Additionally, an exercise-induced increase of CK, LDH, and pain score was observed in both groups (Supplemental Table 1). A maximum increase of LDH was demonstrated three hours after exercise in both groups. In contrast, CK and the pain score peaked at 24 h after exercise in both groups. CK levels, which were one of the primary outcome measures, showed borderline statistical significance (*p* = 0.05) and LDH (*p* = 0.06) with lower values in the Tr14 group (Table 2). Additionally, the subjects were classified into three groups according to their peak CK values: low responders (<500 U/L), medium responders (500–2000 U/L), and high responders (>2000 U/L) (Supplemental Table 2). However, we could not show any differences between placebo and Tr14. Similarly, no group differences were found for either total or VAS pain score or for the strength measurements (extension and flexion) (Table 2).

### 3.3. White Blood Count, Lymphocyte Activation, and Apoptosis

There was an exercise-induced increase of leucocytes in both groups. Thereby, neutrophils and monocytes increased three hours after exercise while lymphocytes increased one hour after exercise and subsequently decreased in both groups (Supplemental Table 1). The Tr14 group indicated less pronounced postexercise lymphopenia (*p* = 0.02) (Table 2). Also, the lymphocyte activation marker CD69 count showed a trend towards a lower level in the Tr14 group (*p* = 0.07) (Table 2). Also, a trend towards lower level neutrocytosis was observed in the Tr14 group (*p* = 0.10) (Table 2). For all other immune cell numbers and apoptosis and surface markers, no treatment effects could be shown (Table 2).

### 3.4. Soluble Inflammatory Mediators

Various anti- (e.g., IL-1ra and IL-10) and proinflammatory (e.g., IL-1*β*, IL-6, and TNF-*α*) cytokines reached their maximum three hours after exercise and subsequently decreased to baseline values within 72 hours in both groups (Supplemental Table 1). However, these mediators showed no statistically significant difference between the two groups. In contrast, other inflammatory mediators such as ICAM-1, IL-18, and IL-12p70 showed group differences. The Tr14 group indicated a lower expression of antigen-stimulated ICAM-1 (*p* = 0.01) (Table 2). Furthermore, antigen-stimulated cytokine IL-18 showed a trend towards a lower level in the Tr14 group (*p* = 0.09) (Table 2). On the contrary, we found a lower decrease of antigen-stimulated IL-12p70 in the verum group (*p* = 0.03) (Table 2). For all other inflammatory mediators, no treatment effects could be shown (Table 2).

### 3.5. Growth Factors

The antigen-stimulated growth factors (BDNF, GM-CSF, IL-3, SCF, and VEGF) were enhanced by exercise in both groups (Supplemental Table 1). In addition, three of the growth factors showed group differences. There was a lower expression of antigen-stimulated BDNF (*p* = 0.03) and a trend towards lower values of antigen-stimulated SCF (*p* = 0.09) in the Tr14 group (Table 2). On the contrary, antigen-stimulated GM-CSF demonstrated a trend towards a higher expression in the Tr14 group (*p* = 0.09) (Table 2). No treatment effects could be found for either IL-3 or VEGF (Table 2).

## 4. Discussion

The result of the current study demonstrated that muscle damage and inflammatory response were slightly modulated by Tr14 after acute bout of eccentric exercise. It also corroborates the previously published results in another strenuous exercise setting [20]. While trends towards lower levels on parameters of muscle damage were observed, Tr14 affected lymphopenia, cellular and soluble activation markers, lowered exercise-induced neutrocytosis, and reduced the expression of selected growth factors such as BDNF. Therefore, Tr14 might limit the destructive processes after eccentric exercise. Relevance for improved regeneration has not been shown in the analyzed parameters. Whether these effects on the cellular recovery in the postexercise period might have any relevance for the adaptational training response remains to be shown.

The eccentric exercise protocol was effective in inducing distinct muscle damage as indicated by a loss of muscle strength and release of intracellular proteins such as CK and LDH in both groups [3, 7]. However, there were no group differences in muscle strength, which is one of the most reliable markers of exercise-induced muscle damage [29]. We assume that the lack of differences is due to the large variability of muscle damage [30]. However, our data indicated a trend towards lower levels of serum muscle damage markers following ingestion of Tr14 after the eccentric exercise suggesting a protective effect on muscle integrity. It can be speculated that this effect could be even more pronounced if Tr14 ingestion had started before and not after the exercise bout. Previous studies focused on individual components of Tr14 on the inflammatory response after exercise [31, 32]. Tr14 consists mostly of components of the Compositae family such as arnica. Application of isolated arnica demonstrated no effects on muscle damage [31, 32]. Lussignoli et al. examined the effect of Tr14 and its components in rats with edema [22]. They found a more rapid decrease of paw edema in rats treated with Tr14, associated with an improved process of healing. Accordingly, it is concluded that the effect of Tr14 seems not to depend on an individual component, but the synergy of all components of Tr14. This may explain that arnica alone did not affect any parameter of muscle damage. Regarding the use of CK, it has to be recognized that a single marker of muscle damage is anyway a limitation of our and other studies because there is a large interindividual variability of CK. Therefore, there is still a discussion on whether CK is a reliable marker for muscle damage [33]. In many studies, the subjects were divided into low, medium, and high responders based on CK activity [34, 35]. Paulsen et al. proposed to make this classification for a better presentation and interpretation [36]. Although the CK response showed high interindividual variations, we cannot conclude any differences from the *p* value between the groups (Supplemental Table 2).

In addition to muscle damage, the consecutive inflammatory process is considered to be a major cause of DOMS [37]. It is known that the expression of various chemokines in damaged muscle tissue is followed by mobilization of leukocytes into the circulation and eventually their infiltration into muscle tissue. In line with previous studies, an exercise-induced increase of circulating leukocytes was found followed by postexercise lymphopenia [37]. It is proposed that lymphocytes initially increase due to redistribution processes from lymphatic and nonlymphatic organs followed by apoptotic cell death and/or redistribution into tissues [38, 39]. In this regard, adhesion molecules such as selectins and ICAM-1 play an important role in transmigration of lymphocytes to the inflammation site [40]. Ingestion of Tr14 induced a lower decrease of lymphocytes, a reduced expression of antigen-stimulated ICAM-1, and a trend towards a lower expression of absolute CD69 in the Tr14 group. Since CD69 is known to stimulate cell proliferation and cytokine secretion, all of these effects of Tr14 are suggested to represent less pronounced activation of the adaptive immune system [41]. Thereby, the lower levels of cell activation and adhesion molecule expression might be responsible for lower emigration of lymphocytes into muscle thereby contributing to the lower lymphopenia. Indirectly, this might also reflect a lower level of damaged muscle tissue.

Besides components of the adaptive immune system, also the innate immune system plays an important role in muscle damage [42]. In accordance with previous studies, eccentric exercise was accompanied by substantial neutrophilia [37]. Here, we showed that the exercise-induced increase of neutrophils was lower by trend after ingestion of Tr14. In general, it is assumed that neutrophils are the first cells which infiltrate the damaged tissue [42]. These cells eliminate cell fragments and performed reorganization processes via reactive oxygen species [43]. The lower peripheral increase of neutrophils in the Tr14 group is the result of lower mobilization into the blood which might be the result of a lower expression of hematopoietic factors [37]. In line with this data, Pilat et al. showed a lower level of neutrophils in the Tr14 group after a concentric exercise test [20]. Possibly, Tr14 affects the number of mobilized neutrophils by inhibition of GM-CSF, which leads to reduced recruitment of neutrophils into circulation. However, such an underlying mechanism could not be confirmed in the current study.

In response to exercise, cells of the innate immune system are also known to produce several proinflammatory cytokines [37]. In this regard, we showed that the proinflammatory antigen-stimulated cytokine IL-12p70 showed a higher level after exercise in the Tr14 group. IL-12 is produced mainly from dendritic cells, monocytes, and macrophages. It plays an important role in the regulation of T helper 1 cell (Th1) responses [44]. A higher level of IL-12p70 indicates a higher type 1 T cell response after exercise. It was demonstrated in a stimulated human whole-blood model that the production of IL-12p70 is inhibited by various stress hormones (dexamethasone, norepinephrine, and epinephrine) [45]. Possibly, Tr14 caused a reduced stress response and lower production of stress hormones resulting in an extenuated T cell mediated immune response. However, no stress hormones were analyzed in this study to support this assumption. Oberbaum et al. demonstrated in an in vivo sepsis model in rats a higher expression of proinflammatory cytokine IL-1*β* in the Tr14 group [23]. Similarly, Pilat et al. demonstrated a higher expression of the proinflammatory cytokines IL-1*β* and IL-1*α* after a concentric exercise test in the Tr14 group [20]. It is suggested that the higher levels of proinflammatory cytokines have a protective effect [23] because a previous injection of IL-1*β* showed improved survival in a murine sepsis model [46]. In contrast, in a study of Porozov et al., a lower expression of proinflammatory cytokines IL-1*β*, TNF-*α*, and CXCL8 in the Tr14 group was found [24]. This study examined the effects of Tr14 in an in vitro model, so the methodological component might have an impact on the results. However, we suggest that the contradictory findings on Tr14 might also be caused by a pleiotropic effect induced by variable effects on different target cells [47].

Altered expression of proinflammatory cytokines might also be due to redox disturbances which are known to affect MAPK and NF-*κ*B signaling [48, 49]. In this regard, Michailidis et al. indicated that N-acetylcysteine lowered proinflammatory response after eccentric exercise [49]. They suggested that the attenuation of NF-*κ*B/MAPK by antioxidants might lower proinflammatory response. However, in the current study, no markers of oxidative stress or related signaling pathways have been analyzed.

It is widely accepted that the exercise-induced muscle damage is part of a muscular adaptation process. This adaptation is visible in the so-called “repeated bout effect,” a term which describes lower level of muscle damage and DOMS after a repeated eccentric exercise bout [50]. In this regard, it is assumed that in response to muscle damage growth factors are expressed which play a role in the regeneration and adaptation of skeletal muscle. In this regard, Menetrey et al. showed that basic fibroblast growth factor (b-FGF), insulin growth factor type 1 (IGF-1), and at a less intensity nerve growth factor (NGF) amplify muscle regeneration in vivo [51]. In general, growth factors play a role in exercise-induced stimulation of cellular growth, proliferation, and differentiation [52]. In our study, we analyzed various growth factors like BDNF, SCF, GM-CSF, IL-3, and VEGF. BDNF belongs to the neuropathic family as well as NGF [53]. In the Tr14 group, a lower expression of BDNF and a trend towards lower expression of SCF were found. There is some evidence that BDNF plays a role during myogenic differentiation if it is expressed by satellite cells [54]. Similarly, SCF is suggested to be involved in the stimulation of muscle-derived stem cell [55] and plays a major role in hematopoiesis [56]. The reduced expression of BDNF and SCF in the Tr14 group might indicate a reduced need for tissue regeneration. However, if the reduction of muscle damage is the reason for lower growth factors, expression remains to be shown.

For the interpretation of the current results, it has to be considered that a stimulated in vitro whole-blood culture was used for analysis of the cytokines and growth factors. An advantage of using whole-blood culture is that the cellular environment is similar to in vivo blood conditions [57]. However, the included stimulants LPS and SEB stimulate primarily monocytes and lymphocytes, respectively [58, 59]. This means that the effects are primarily dependent on these cell types. The complete synergy of the cytokines in all compartments is not shown in this model, so that no conclusions can be concluded about the exact mechanism of the effect of Tr14.

In our study, Tr14 was ingested after the exercise bout. Possibly, the ingestion of Tr14 before the eccentric exercise may have a more pronounced preventive effect. Accordingly, further studies including the analysis of detailed mechanisms of the effects of Tr14 still need to be performed. Besides this, a potential negative effect of anti-inflammatory drugs on adaptation processes has to be considered and investigated in future studies.

In summary, these results indicate that Tr14 affects exercise-induced muscle damage leading to reduced activation of the innate and the adaptive immune system in response to eccentric exercise. Possibly, these anti-inflammatory actions accelerate the regeneration processes. However, any practical impact for athletes remains to be shown.

## Supplementary Material

The Supplementary Material consists of two tables. Supplemental table S1 shows the results of all outcome parameters (Median, 1. and 3. quartile) in placebo and Tr14 group. Supplemental table S2 demonstrates the classification of the CK response (low, medium, high).

## Figures and Tables

**Table 1:** Subject characteristics of placebo and Tr14 group (the median with the 1st and 3rd quartile is shown in parentheses).

	Placebo (*n* = 47)	Tr14 (*n* = 48)	*p* value
Age (years)	25.0 (23.0; 28.0)	24.0 (21.0; 27.0)	0.20
Height (cm)	183.0 (176.0; 187.0)	182.0 (176.5; 186.5)	0.75
Weight (kg)	79.4 (72.5; 84.0)	76.8 (67.9; 86.3)	0.73
BMI (kg/m^2^)	23.8 (21.9; 25.4)	23.7 (21.6; 25.7)	0.64
VO_2max_ (mL/min/kg)	43.9 (39.7; 47.3)	45.7 (42.8; 48.9)	0.20
HR at 80% VO_2max_ (beats/min)	161.0 (152.0; 170.0)	164.5 (153.5; 174.0)	0.29

BMI: body mass index; HR: heart rate; VO_2max_: maximum oxygen uptake.

Median with the 1st and 3rd quartile in parentheses.

**Table 2:** AUC_i_ median values from all outcome parameters and the *p* value of median test.

	AUC_i_ median values		*p* value
	Placebo	Tr14	
*Muscle damage and recovery*			
CK	1.7 × 10^6^	1.1 × 10^6^	0.05
LDH	1.0 × 10^5^	6.1 × 10^4^	0.06
Strength (extension)	−2.7 × 10^5^	−2.2 × 10^5^	0.87
Strength (flexion)	−1.2 × 10^5^	−8.7 × 10^4^	0.30
Pain score, total	1.2 × 10^5^	1.4 × 10^5^	0.36
Pain score, VAS	9.3 × 10^4^	1.1 × 10^5^	0.36
*White blood cell count*			
Basophils	0	0	0.85
Basophils absolute	−5.4	−6.9	0.41
Eosinophils	−1.2 × 10^3^	−2.2 × 10^3^	0.41
Eosinophils absolute	−58.3	−66.0	0.41
Leukocytes	2.2 × 10^3^	3.4 × 10^3^	0.92
*Lymphocytes*	*−1.7 × 10* ^*4*^	*−7.9 × 10* ^*3*^	*0.02*
Lymphocytes absolute	−3.9 × 10^2^	−1.2 × 10^2^	0.21
Monocytes	−4.1 × 10^2^	−4.0 × 10^3^	0.21
Monocytes absolute	1.4 × 10^2^	63.2	0.41
Neutrophils	2.0 × 10^4^	1.4 × 10^4^	0.21
Neutrophils absolute	3.4 × 10^3^	2.3 × 10^3^	0.10
*Lymphocyte activation and apoptosis*			
Annexin V	−1.9 × 10^3^	−3.7 × 10^3^	0.67
Annexin V absolute	−44.5	−66.2	0.92
CD62	4.1 × 10^3^	−4.7 × 10^3^	0.21
CD62L absolute	−2.9 × 10^2^	−1.6 × 10^2^	0.60
CD69	1.1 × 10^2^	2.8 × 10^2^	0.41
CD69 absolute	−0.4	5.4	0.07
CD95R	3.1 × 10^3^	5.9 × 10^3^	0.18
CD95R absolute	−0.8	36.2	0.20
CD95L	−5.5 × 10^2^	5.1 × 10^3^	0.47
CD95L absolute	−7.0	40.5	0.34
*Soluble inflammatory mediators*			
CXCL8	4.9 × 10^7^	3.0 × 10^7^	0.40
CXCL8 serum	1.2 × 10^3^	1.9 × 10^3^	0.60
CCL2	8.6 × 10^6^	4.0 × 10^7^	0.40
CCL2 serum	1.4 × 10^5^	1.3 × 10^5^	0.61
CCL3	5.0 × 10^7^	3.8 × 10^7^	0.40
CCL4	2.7 × 10^8^	3.4 × 10^8^	0.67
CCL11	−1.3 × 10^4^	−6.3 × 10^4^	0.20
Factor VII	2.5 × 10^4^	−3.8 × 10^4^	0.40
*ICAM-1*	*1.3 × 10* ^*4*^	*−2.9 × 10* ^*3*^	*0.01*
IFN-*γ*	2.6 × 10^6^	2.3 × 10^6^	0.67
IL-1*α*	2.3 × 10^2^	2.6 × 10^2^	0.67
IL-1*β*	1.4 × 10^7^	9.0 × 10^6^	0.40
IL-1*β* serum	3.8 × 10^2^	3.2 × 10^−7^	0.46
IL-1ra	4.5 × 10^6^	4.5 × 10^6^	1.00
IL-1ra serum	−4.8 × 10^4^	1.8 × 10^4^	0.34
IL-2	−2.4 × 10^5^	−4.0 × 10^4^	0.40
IL-4	−3.0 × 10^4^	3.8 × 10^4^	0.67
IL-5	1.0 × 10^3^	4.6 × 10^3^	0.20
IL-6	3.8 × 10^7^	2.9 × 10^7^	0.67
IL-6 serum	0	0	0.50
IL-7	4.0 × 10^4^	3.5 × 10^3^	0.67
IL-10	1.9 × 10^5^	2.1 × 10^4^	0.40
IL-12p40	2.3 × 10^3^	5.0 × 10^3^	0.67
*IL-12p70*	*−8.6 × 10* ^*4*^	*1.7 × 10* ^*4*^	*0.03*
IL-15	6.1 × 10^2^	5.0 × 10^2^	0.40
IL-17	−5.4 × 10^3^	−3.9 × 10^3^	1.00
IL-18	2.0 × 10^5^	1.0 × 10^5^	0.09
IL-23	2.8 × 10^3^	5.9 × 10^3^	0.67
TNF-*α*	1.2 × 10^7^	1.1 × 10^7^	1.00
TNF-*α* serum	0	0	0.58
TNF-*β*	5.9 × 10^3^	6.0 × 10^3^	1.00
*Growth factors*			
*BDNF*	*4.5 × 10* ^*3*^	*−1.9 × 10* ^*2*^	*0.03*
GM-CSF	2.0 × 10^4^	7.5 × 10^4^	0.09
IL-3	9.4	0.8	0.40
SCF	5.7 × 10^5^	2.4 × 10^5^	0.09
VEGF	1.2 × 10^5^	8.1 × 10^4^	0.40
*Matrix metalloproteinases*			
MMP-3	5.3 × 10^3^	3.9 × 10^3^	0.20
MMP-9	6.3 × 10^4^	5.0 × 10^4^	0.40
*Acute phase protein*			
CRP	2.4 × 10^3^	2.3 × 10^3^	0.76

BDNF: brain-derived neurotrophic factor; CK: creatine kinase; CRP: C-reactive protein; GM-CSF: granulocyte macrophage colony-stimulating factor; ICAM-1: intercellular adhesion molecule 1; IFN-*γ*: interferon-gamma; LDH: lactate dehydrogenase; MMP: matrix metalloproteinase; SCF: stem cell factor; TNF: tumor necrosis factor; VEGF: vascular endothelial growth factor.

## References

[1] Walsh N. P., Gleeson M., Shephard R. J. (2011). Position statement part one: immune function and exercise. *Exercise Immunology Review*.

[2] Armstrong R. B. (1984). Mechanisms of exercise-induced delayed onset muscular soreness: a brief review. *Medicine and Science in Sports and Exercise*.

[3] MacIntyre D. L., Reid W. D., McKenzie D. C. (1995). Delayed muscle soreness. The inflammatory response to muscle injury and its clinical implications. *Sports Medicine*.

[4] Pyne D. B. (1994). Exercise-induced muscle damage and inflammation: a review. *Australian Journal of Science and Medicine in Sport*.

[5] Fridén J., Sjöström M., Ekblom B. (1983). Myofibrillar damage following intense eccentric exercise in man. *International Journal of Sports Medicine*.

[6] Fridén J., Lieber R. L. (1992). Structural and mechanical basis of exercise-induced muscle injury. *Medicine and Science in Sports and Exercise*.

[7] Peake J. M., Suzuki K., Wilson G. (2005). Exercise-induced muscle damage, plasma cytokines, and markers of neutrophil activation. *Medicine and Science in Sports and Exercise*.

[8] Suzuki K., Nakaji S., Yamada M., Totsuka M., Sato K., Sugawara K. (2002). Systemic inflammatory response to exhaustive exercise. Cytokine kinetics. *Exercise Immunology Review*.

[9] Donnelly A. E., Maughan R. J., Whiting P. H. (1990). Effects of ibuprofen on exercise-induced muscle soreness and indices of muscle damage. *British Journal of Sports Medicine*.

[10] Nieman D. C., Henson D. A., Dumke C. L. (2006). Ibuprofen use, endotoxemia, inflammation, and plasma cytokines during ultramarathon competition. *Brain, Behavior, and Immunity*.

[11] Theodorou A. A., Nikolaidis M. G., Paschalis V. (2011). No effect of antioxidant supplementation on muscle performance and blood redox status adaptations to eccentric training. *American Journal of Clinical Nutrition*.

[12] Lund H., Vestergaard-Poulsen P., Kanstrup I.-L., Sejrsen P. (1998). The effect of passive stretching on delayed onset muscle soreness, and other detrimental effects following eccentric exercise. *Scandinavian Journal of Medicine and Science in Sports*.

[13] Crystal N. J., Townson D. H., Cook S. B., Laroche D. P. (2013). Effect of cryotherapy on muscle recovery and inflammation following a bout of damaging exercise. *European Journal of Applied Physiology*.

[14] Hasson S. M., Daniels J. C., Divine J. G. (1993). Effect of ibuprofen use on muscle soreness, damage, and performance: a preliminary investigation. *Medicine and Science in Sports and Exercise*.

[15] O'Grady M., Hackney A. C., Schneider K. (2000). Diclofenac sodium (Voltaren) reduced exercise-induced injury in human skeletal muscle. *Medicine and Science in Sports and Exercise*.

[16] Hernández-Díaz S., García-Rodríguez L. A. (2001). Epidemiologic assessment of the safety of conventional nonsteroidal anti-inflammatory drugs. *American Journal of Medicine*.

[17] Srivastava J. K., Pandey M., Gupta S. (2009). Chamomile, a novel and selective COX-2 inhibitor with anti-inflammatory activity. *Life Sciences*.

[18] Raso G. M., Pacilio M., Di Carlo G., Esposito E., Pinto L., Meli R. (2002). In-vivo and in-vitro anti-inflammatory effect of Echinacea purpurea and Hypericum perforatum. *Journal of Pharmacy and Pharmacology*.

[19] González de Vega C., Speed C., Wolfarth B., González J. (2013). Traumeel vs. diclofenac for reducing pain and improving ankle mobility after acute ankle sprain: a multicentre, randomised, blinded, controlled and non-inferiority trial. *International Journal of Clinical Practice*.

[20] Pilat C., Frech T., Wagner A. (2015). Exploring effects of a natural combination medicine on exercise-induced inflammatory immune response: a double-blind RCT. *Scandinavian Journal of Medicine and Science in Sports*.

[21] Toliopoulos I. K., Simos Y., Bougiouklis D., Oikonomidis S. (2013). Stimulation of natural killer cells by homoeopathic complexes: an in vitro and in vivo pilot study in advanced cancer patients. *Cell Biochemistry and Function*.

[22] Lussignoli S., Bertani S., Metelmann H., Bellavite P., Conforti A. (1999). Effect of Traumeel S^®^, a homeopathic formulation, on blood induced inflammation in rats. *Complementary Therapies in Medicine*.

[23] Oberbaum M., Spira R. M., Lukasiewicz E. (2011). Effect of traumeel S on cytokine profile in a cecal ligation and puncture (CLP) sepsis model in rats. *Journal of Alternative and Complementary Medicine*.

[24] Porozov S., Cahalon L., Weiser M., Branski D., Lider O., Oberbaum M. (2004). Inhibition of IL-1*β* and TNF-*α* secretion from resting and activated human immunocytes by the homeopathic medication Traumeel^®^ S. *Clinical and Developmental Immunology*.

[25] Schneider C. (2011). Traumeel—an emerging option to nonsteroidal anti-inflammatory drugs in the management of acute musculoskeletal injuries. *International Journal of General Medicine*.

[26] Mooren F. C., Lechtermann A., Völker K. (2004). Exercise-induced apoptosis of lymphocytes depends on training status. *Medicine and Science in Sports and Exercise*.

[27] Melzack R. (1987). The short-form McGill pain questionnaire. *Pain*.

[28] Pruessner J. C., Kirschbaum C., Meinlschmid G., Hellhammer D. H. (2003). Two formulas for computation of the area under the curve represent measures of total hormone concentration versus time-dependent change. *Psychoneuroendocrinology*.

[29] Damas F., Nosaka K., Libardi C., Chen T., Ugrinowitsch C. (2016). Susceptibility to exercise-induced muscle damage: a cluster analysis with a large sample. *International Journal of Sports Medicine*.

[30] Margaritelis N. V., Kyparos A., Paschalis V. (2014). Reductive stress after exercise: the issue of redox individuality. *Redox Biology*.

[31] Plezbert J. A., Burke J. R. (2005). Effects of the homeopathic remedy arnica on attenuating symptoms of exercise-induced muscle soreness. *Journal of Chiropractic Medicine*.

[32] Pumpa K. L., Fallon K. E., Bensoussan A., Papalia S. (2014). The effects of topical Arnica on performance, pain and muscle damage after intense eccentric exercise. *European Journal of Sport Science*.

[33] Koch A. J., Pereira R., Machado M. (2014). The creatine kinase response to resistance exercise. *Journal of Musculoskeletal Neuronal Interactions*.

[34] Machado M., Pereira R., Willardson J. M. (2012). Short intervals between sets and individuality of muscle damage response. *Journal of Strength and Conditioning Research*.

[35] Kim J., Lee J. (1975). The relationship of creatine kinase variability with body composition and muscle damage markers following eccentric muscle contractions. *The Journal of Exercise Nutrition and Biochemistry*.

[36] Paulsen G., Mikkelsen U. R., Raastad T., Peake J. M. (2012). Leucocytes, cytokines and satellite cells: what role do they play in muscle damage and regeneration following eccentric exercise?. *Exercise Immunology Review*.

[37] Peake J., Nosaka K., Suzuki K. (2005). Characterization of inflammatory responses to eccentric exercise in humans. *Exercise Immunology Review*.

[38] Krüger K., Lechtermann A., Fobker M., Völker K., Mooren F. C. (2008). Exercise-induced redistribution of T lymphocytes is regulated by adrenergic mechanisms. *Brain, Behavior, and Immunity*.

[39] Krüger K., Mooren F. C. (2014). Exercise-induced leukocyte apoptosis. *Exercise Immunology Review*.

[40] Krüger K., Mooren F. C. (2007). T cell homing and exercise. *Exercise Immunology Review*.

[41] Sancho D., Gómez M., Sánchez-Madrid F. (2005). CD69 is an immunoregulatory molecule induced following activation. *Trends in Immunology*.

[42] Clarkson P. M., Sayers S. P. (1999). Etiology of exercise-induced muscle damage. *Canadian Journal of Applied Physiology*.

[43] Butterfield T. A., Best T. M., Merrick M. A. (2006). The dual roles of neutrophils and macrophages in inflammation: a critical balance between tissue damage and repair. *Journal of Athletic Training*.

[44] Langrish C. L., McKenzie B. S., Wilson N. J., de Waal Malefyt R., Kastelein R. A., Cua D. J. (2004). IL-12 and IL-23: master regulators of innate and adaptive immunity. *Immunological Reviews*.

[45] Elenkov I. J., Papanicolaou D. A., Wilder R. L., Chrousos G. P. (1996). Modulatory effects of glucocorticoids and catecholamines on human interleukin-12 and interleukin-10 production: clinical implications. *Proceedings of the Association of American Physicians*.

[46] Michael O., Silver G. M., Davis J. H., Gamelli R. L., Hebert J. C. (1992). Interleukin 1*β* improves survival following cecal ligation and puncture. *Journal of Surgical Research*.

[47] Ozaki K., Leonard W. J. (2002). Cytokine and cytokine receptor pleiotropy and redundancy. *The Journal of Biological Chemistry*.

[48] Tidball J. G., Villalta S. A. (2010). Regulatory interactions between muscle and the immune system during muscle regeneration. *American Journal of Physiology-Regulatory Integrative and Comparative Physiology*.

[49] Michailidis Y., Karagounis L. G., Terzis G. (2013). Thiol-based antioxidant supplementation alters human skeletal muscle signaling and attenuates its inflammatory response and recovery after intense eccentric exercise. *The American Journal of Clinical Nutrition*.

[50] Mair J., Mayr M., Muller E. (1995). Rapid adaptation to eccentric exercise-induced muscle damage. *International Journal of Sports Medicine*.

[51] Menetrey J., Kasemkijwattana C., Day C. S. (2000). Growth factors improve muscle healing in vivo. *The Journal of Bone & Joint Surgery—British Volume*.

[52] Goustin A. S., Leof E. B., Shipley G. D., Moses H. L. (1986). Growth factors and cancer. *Cancer Research*.

[53] Binder D. K., Scharfman H. E. (2004). Brain-derived neurotrophic factor. *Growth Factors*.

[54] Mousavi K., Jasmin B. J. (2006). BDNF is expressed in skeletal muscle satellite cells and inhibits myogenic differentiation. *The Journal of Neuroscience*.

[55] Deasy B. M., Qu-Peterson Z., Greenberger J. S., Huard J. (2002). Mechanisms of muscle stem cell expansion with cytokines. *Stem Cells*.

[56] Broudy V. C. (1997). Stem cell factor and hematopoiesis. *Blood*.

[57] Schmolz M., Hurst T. L., Bailey D. M. (2004). Validation of a new highly standardised, lab-independent whole-blood leukocyte function assay for clinical trials (ILCS^®^). *Experimental Gerontology*.

[58] Guha M., Mackman N. (2001). LPS induction of gene expression in human monocytes. *Cellular Signalling*.

[59] Krakauer T. (2013). Update on staphylococcal superantigen-induced signaling pathways and therapeutic interventions. *Toxins*.

