# Supplementary material for: Effects of Traumeel (Tr14) on Exercise-Induced Muscle Damage Response in Healthy Subjects: A Double-Blind RCT
Source: Mediators Inflamm. 2016 Jul 5;2016:1693918. doi: 10.1155/2016/1693918 (PMC4949332; doi:10.1155/2016/1693918)
Supplement: Supplementary file 1 — The Supplementary Material consists of two tables. Supplemental table S1 shows the results of all outcome parameters (Median, 1. and 3. quartile) in placebo and Tr14 group. Supplemental table S2 demonstrates the classification of the CK response (low, medium, high). [file 1693918.f1.pdf]

Supplemental table S1

	gr	pre		post		3 h post		24 h post		48 h post		72 h post	
Muscle damage and recovery													
CK	Pl	1.3	(1.0; 2.1)	2.0	(1.5; 2.7)	4.9	(3.3; 6.0)	7.8	(4.6; 1.2)	4.3	(2.5; 5.8)	3.0	(2.0; 4.4)
[U/L] x10^2	Tr	1.3	(1.0; 2.2)	1.9	(1.5; 3.6)	4.0	(3.2; 5.9)	6.5	(4.6; 10.0)	3.9	(2.7; 5.8)	2.7	(1.7; 4.7)
LDH	Pl	1.6	(1.5; 1.9)	2.1	(1.9; 2.2)	2.2	(1.9; 2.3)	1.9	(1.8; 2.1)	1.8	(1.7; 2.0)	1.9	(1.7; 2.0)
[U/L] x10^2	Tr	1.8	(1.6; 2.0)	2.2	(2.0; 2.3)	2.2	(2.0; 2.4)	1.9	(1.7; 2.2)	1.8	(1.7; 2.0)	1.8	(1.6; 2.0)
strength (Extension)	Pl	4.0	(3.3; 4.6)	3.1	(2.6; 3.9)	3.0	(2.6; 3.6)	3.1	(2.4; 3.6)	3.2	(2.5; 3.7)	2.5	(2.9; 4.1)
[Nm] x10^2	Tr	3.8	(3.3; 4.3)	3.0	(2.5; 3.6)	3.0	(2.6; 3.9)	3.3	(2.3; 3.9)	3.4	(2.8; 4.0)	3.5	(3.0; 4.4)
strength (Flexion)	Pl	1.9	(1.7; 2.1)	1.6	(1.3; 1.8)	1.7	(1.3; 1.9)	1.5	(1.1; 1.8)	1.6	(1.2; 1.9)	1.6	(1.4; 2.0)
[Nm] x10^2	Tr	1.8	(1.5; 2.2)	1.5	(1.3; 1.9)	1.5	(1.2; 1.9)	1.5	(1.3; 1.9)	1.6	(1.4; 1.8)	1.7	(1.4; 2.1)
Pain Score Total	Pl	0	(0; 3.0)	32.0	(17.0; 49.0)	26.0	(11.0; 37.0)	41.0	(27.0; 65.0)	36.0	(19.0; 53.0)	14.0	(6.0; 25.0)
	Tr	0	(0; 1.0)	30.0	(18.5; 50.0)	28.5	(13.0; 44.5)	41.0	(25.5; 54.0)	36.0	(17.0; 57.0)	17.5	(8.0; 26.0)
Pain Score VAS	Pl	0	(0; 2.0)	22.0	(9.0; 36.0)	16.0	(6.0; 28.0)	32.0	(19.0; 52.0)	25.0	(15.0; 38.0)	10.0	(3.0; 18.0)
	Tr	0	(0; 1.0)	24.0	(9.0; 33.0)	22.0	(8.5; 31.5)	31.5	(19.0; 40.0)	27.5	(13.5; 43.5)	13.0	(5.5; 20.5)
White blood cell count													
Basophile	Pl	0.1	(0; 1.0)	0.3	(0; 1.0)	0	(0; 0.2)	0	(0; 1.0)	0.2	(0; 1.0)	0.2	(0; 1.0)
[%]	Tr	0.3	(0; 1.0)	0.6	(0; 1.0)	0	(0; 0.2)	0.2	(0; 1.0)	0.2	(0; 1.0)	0.4	(0; 1.0)
Basophile absolute	Pl	2.0	(2.0; 3.0)	3.0	(2.0; 4.0)	2.0	(2.0; 3.0)	2.0	(1.0; 3.0)	2.2	(2.0; 3.0)	2.0	(1.0; 3.0)
[c/nl] x10^{-2}	Tr	2.0	(2.0; 3.0)	3.0	(2.0; 4.0)	2.0	(2.0; 3.0)	2.2	(2.0; 3.0)	2.0	(1.0; 3.0)	2.0	(2.0; 3.0)
Eosinophils	Pl	3.0	(2.0; 4.0)	2.0	(1.0; 3.0)	1.0	(1.0; 1.3)	3.0	(2.0; 4.0)	2.6	(2.0; 4.0)	2.3	(2.0; 4.0)
[%]	Tr	3.0	(2.0; 4.3)	2.0	(1.0; 3.0)	1.0	(1.0; 2.0)	3.0	(2.0; 4.1)	3.0	(2.0; 4.0)	3.0	(2.0; 4.0)
Eosinophils absolute	Pl	0.2	(0.1; 0.2)	0.1	(0.1; 0.2)	0.1	(0.1; 0.1)	0.1	(0.1; 0.2)	0.2	(0.1; 0.2)	0.1	(0.1; 0.2)
[c/nl]	Tr	0.2	(0.1; 0.2)	0.1	(0.1; 0.2)	0.1	(0.1; 0.2)	0.2	(0.1; 0.3)	0.1	(0.1; 0.2)	0.1	(0.1; 0.2)
Leukocytes	Pl	5.4	(4.6; 6.6)	6.8	(5.5; 7.8)	8.8	(7.5; 10.2)	5.6	(4.5; 6.5)	5.4	(4.6; 6.2)	5.3	(4.4; 5.9)
[tsd/µl]	Tr	5.4	(4.8; 6.3)	6.5	(5.4; 7.5)	8.4	(7.8; 10.2)	5.4	(4.8; 6.6)	5.2	(4.5; 5.9)	5.2	(4.7; 6.0)
Lymphocytes	Pl	34.9	(30.0; 39.0)	33.5	(29.0; 39.0)	20.0	(17.0; 27.0)	31.0	(26.0; 37.0)	33.0	(27.0; 37.0)	32.7	(28.0; 38.0)
[%]	Tr	32.0	(28.0; 37.6)	31.1	(27.0; 35.2)	22.0	(18.3; 26.0)	32.0	(27.0; 36.3)	31.3	(29.0; 36.0)	31.0	(28.0; 35.4)
Lymphocytes absolute	Pl	1.9	(1.5; 2.3)	2.2	(1.7; 2.5)	1.8	(1.5; 2.2)	1.8	(1.4; 2.1)	1.7	(1.5; 2.0)	1.7	(1.4; 2.0)
[c/nl]	Tr	1.7	(1.5; 2.1)	2.0	(1.6; 2.6)	1.9	(1.7; 2.1)	1.7	(1.5; 2.0)	1.7	(1.4; 1.9)	1.6	(1.4; 1.9)
Monocytes	Pl	8.0	(7.0; 9.4)	8.0	(7.0; 9.0)	8.0	(6.0; 9.0)	8.0	(7.0; 9.1)	8.3	(7.0; 10.0)	8.0	(7.0; 10.0)
[%]	Tr	9.0	(7.4; 10.0)	8.0	(7.0; 10.0)	8.0	(7.0; 9.0)	8.0	(7.0; 10.0)	8.7	(7.3; 10.0)	8.0	(7.0; 10.0)
Monocytes absolute	Pl	0.4	(0.4; 0.5)	0.6	(0.4; 0.7)	0.6	(0.5; 0.8)	0.4	(0.4; 0.5)	0.5	(0.4; 0.5)	0.4	(0.3; 0.5)
[c/nl]	Tr	0.5	(0.4; 0.6)	0.5	(0.4; 0.6)	0.7	(0.6; 0.9)	0.4	(0.4; 0.6)	0.5	(0.4; 0.5)	0.4	(0.4; 0.5)
Neutrophils	Pl	54.0	(49.5; 57.0)	55.0	(52. 0; 61.0)	71.0	(64.0; 74.0)	56.7	(51.0; 62.0)	57.0	(50.0; 61.0)	57.0	(50.0; 60.0)
[%]	Tr	56.9	(49.5; 60.0)	59.0	(51.4; 63.0)	67.5	(63.5; 72.1)	55.1	(52.0; 61.8)	55.2	(50.9; 60.1)	57.0	(49.0; 62.0)
Neutrophils absolute	Pl	2.8	(2.4; 3.4)	3.7	(3.1; 4.3)	5.8	(5.0; 7.2)	3.2	(2.4; 3.7)	2.9	(2.4; 3.6)	2.8	(2.5; 3.4)
[c/nl]	Tr	3.2	(2.3; 3.6)	3.7	(3.1; 4.4)	5.8	(4.9; 7.2)	3.1	(2.5; 3.8)	2.8	(2.5; 3.6)	3.0	(2.5; 3.7)

Lymphocyte activation and apoptosis													
Annexin V	Pl	7.2	(6.3; 10.4)	7.6	(5.7; 10.5)	6.1	(4.2; 8.4)	7.0	(5.8; 9.0)	6.8	(5.2; 8.5)	6.8	(5.1; 10.9)
[%]	Tr	7.6	(5.6; 9.7)	6.8	(5.5; 10.4)	5.8	(4.3; 9.2)	6.7	(5.2; 9.0)	7.5	(5.3; 10.2)	6.6	(4.9; 10.7)
Annexin V absolute	Pl	1.3	(1.0; 1.9)	1.6	(1.2; 2.2)	1.1	(0.7; 1.6)	1.1	(0.9; 1.6)	1.1	(0.9; 1.6)	1.1	(0.7; 2.0)
[x10^6/ml] x10^{-1}	Tr	1.3	(0.9; 2.1)	1.4	(0.9; 2.0)	1.1	(0.7; 1.9)	1.1	(0.9; 1.8)	1.2	(0.9; 1.8)	1.1	(0.8; 1.7)
CD62L	Pl	66.2	(55.8; 73.7)	61.8	(50.0; 68.7)	68.2	(58.0; 74.3)	65.4	(57.3; 70.8)	67.3	(57.5; 70.8)	66.1	(57.5; 70.7)
[%]	Tr	65.2	(60.3; 76.9)	63.8	(56.7; 68.9)	69.6	(61.7; 74.8)	63.9	(56.8; 70.8)	65.6	(60.8; 72.0)	66.9	(60.2; 73.5)
CD62L absolute	Pl	1.3	(0.8; 1.6)	1.4	(1.0; 1.7)	1.2	(0.9; 1.5)	1.1	(0.8; 1.3)	1.2	(0.8; 1.4)	1.2	(0.8; 1.4)
[x10^6/ml]	Tr	1.1	(0.9; 1.5)	1.2	(1.0; 1.7)	1.4	(1.1; 1.5)	1.1	(0.8; 1.5)	1.1	(0.9; 1.3)	1.0	(0.9; 1.3)
CD69	Pl	0.4	(0.2; 0.7)	0.4	(0.2; 0.6)	0.4	(0.2; 0.6)	0.5	(0.2; 0.7)	0.4	(0.3; 0.6)	0.5	(0.3; 0.7)
[%]	Tr	0.3	(0.2; 0.7)	0.5	(0.3; 0.8)	0.4	(0.2; 0.7)	0.4	(0.2; 0.6)	0.4	(0.2; 0.6)	0.4	(0.2; 0.6)
CD69 absolute	Pl	6.9	(3.9; 11.3)	7.1	(3.8; 14.4)	6.6	(3.2; 10.7)	8.8	(3.3; 18.7)	6.7	(4.0; 13.1)	6.9	(3.6; 11.7)
[x10^6/ml] x10^{-3}	Tr	5.7	(4.0; 12.7)	9.5	(4.1; 17.2)	7.2	(4.0; 13.0)	6.4	(3.4; 11.1)	7.7	(4.1; 11.6)	5.1	(2.6; 10.0)
CD95R	Pl	15.7	(13.0; 20.4)	14.0	(11.8; 20.2)	16.4	(13.8; 20.1)	15.7	(13.2; 21.5)	16.4	(13.4; 22.2)	16.9	(13.3; 20.3)
[%]	Tr	16.0	(13.5; 20.1)	16.6	(14.3; 19.7)	18.5	(15.2; 21.5)	17.6	(13.4; 20.8)	17.9	(15.0; 21.0)	18.2	(15.0; 22.1)
CD95R absolute	Pl	0.3	(0.2; 0.4)	0.3	(0.2; 0.4)	0.3	(0.2; 0.4)	0.3	(0.2; 0.4)	0.3	(0.2; 0.4)	0.4	(0.2; 0.4)
[x10^6/ml]	Tr	0.3	(0.2; 0.4)	0.3	(0.3; 0.4)	0.4	(0.3; 0.5)	0.3	(0.2; 0.4)	0.3	(0.2; 0.4)	0.3	(0.2; 0.4)
CD95L [%]	Pl	6.3	(3.8; 11.4) [n=38]	3.8	(2.6; 6.1) [n=39]	4.9	(1.7; 9.2) [n=39]	6.5	(2.3; 11.3) [n=39]	4.7	(2.1; 8.3) [n=39]	5.1	(2.6; 12.9) [n=39]
[batch 1]	Tr	5.2	(2.9; 12.7) [n=39]	3.9	(2.7; 6.1) [n=40]	4.9	(2.2; 8.6) [n=40]	4.2	(2.9; 11.3) [n=39]	6.6	(2.7; 18.5) [n=40]	3.5	(1.8; 10.4) [n=39]
CD95L [%]	Pl	67.2	(53.1; 77.8) [n=8]	80.0	(70.0; 86.8) [n=8]	72.2	(61.3; 90.5) [n=8]	76.3	(62.0; 81.7) [n=8]	66.6	(48.3; 86.7) [n=8]	79.4	(56.3; 89.5) [n=8]
[batch 2]	Tr	68.3	(52.0; 74.1) [n=7]	72.5	(68.0; 76.9) [n=7]	66.8	(48.6; 71.3) [n=7]	71.4	(56.1; 89.2) [n=7]	78.2	(50.2; 93.5) [n=7]	80.0	(56.0; 91.0) [n=7]
CD95L absolute [x10^6/ml] x10^{-1}	Pl	1.3	(0.7; 2.0) [n=37]	0.9	(0.6; 1.7) [n=38]	0.9	(0.4; 1.7) [n=39]	1.0	(0.4; 1.6) [n=39]	0.7	(0.4; 1.4) [n=39]	0.8	(0.4; 1.7) [n=39]
[batch 1]	Tr	0.9	(0.5; 2.1) [n=39]	0.8	(0.6; 1.4) [n=39]	0.8	(0.4; 1.6) [n=40]	0.8	(0.4; 2.6) [n=39]	1.1	(0.5; 3.0) [n=40]	0.7	(0.3; 1.5) [n=39]
CD95L absolute [x10^6/ml]	Pl	1.5	(1.1; 1.6) [n=8]	1.7	(1.5; 1.9) [n=[n=8]	1.4	(1.2; 1.8) [n=8]	1.4	(1.3; 1.8) [n=8]	1.2	(0.9; 1.8) [n=8]	1.5	(1.0; 1.7) [n=8]
[batch 2]	Tr	1.2	(0.9; 1.5) [n=7]	1.3	(1.2; 1.7) [n=7]	1.2	(1.0; 1.4) [n=7]	1.0	(0.9; 1.6) [n=7]	1.2	(0.8; 1.76) [n=7]	1.2	(0.8; 1.7) [n=7]
Soluble inflammatory mediators													
CXCL8	Pl	4.5	(3.2; 6.3)	8.7	(6.1; 10.3)	7.8	(5.2; 12.5)	5.4	(3.1; 7.2)	5.0	(3.9; 6.7)	5.1	(3.1; 7.2)
[pg/ml] x10^4	Tr	5.0	(3.5; 7.1)	8.7	(5.8; 10.6)	8.4	(5.9; 12.5)	4.9	(3.3; 6.6)	4.7	(3.4; 7.3)	5.4	(3.2; 6.9)
CXCL8 serum	Pl	5.5	(4.0; 7.9)	6.3	(4.5; 8.3)	6.1	(4.2; 8.2)	5.7	(4.6; 7.5)	5.6	(4.6; 7.4)	6.1	(4.2; 8.4)
[pg/ml]	Tr	4.9	(3.8; 6.8)	6.5	(4.6; 8.2)	5.6	(4.1; 7.2)	5.6	(4.3; 7.0)	5.3	(3.9; 6.9)	5.5	(3.9; 6.5)
CCL2	Pl	12.7	(6.7; 18.0)	24.1	(17.4; 29.6)	23.4	(13.8; 29.2)	12.9	(7.1; 19.3)	11.4	(9.3; 18.3)	10.1	(6.3; 15.8)
[pg/ml] x10^3	Tr	15.9	(10.5; 21.2)	28.5	(16.8; 40.8)	24.4	(14.7; 35.3)	14.4	(7.7; 21.7)	12.3	(8.7; 21.9)	13.0	(8.3; 19.7)

CCL2 serum	Pl	3.4	(2.7; 4.4)	4.3	(3.7; 5.2)	5.7	(4.5; 7.9)	3.4	(2.8; 4.3)	3.0	(2.6; 3.8)	3.1	(2.6; 3.8)
[pg/ml] x10^{2}	Tr	3.5	(2.9; 5.3)	4.5	(3.8; 6.1)	5.8	(4.6; 7.6)	3.9	(3.1; 4.5)	3.3	(2.9; 4.0)	3.3	(2.5; 4.0)
CCL3	Pl	7.7	(5.7; 10.2)	11.5	(8.9; 14.7)	13.2	(8.6; 19.0)	8.1	(5.3; 10.4)	7.6	(5.8; 11.4)	7.9	(6.2; 10.3)
[pg/ml] x10^{4}	Tr	8.5	(6.2; 10.7)	11.8	(8.8; 14.9)	13.5	(10.6; 16.5)	8.8	(5.8; 11.4)	8.5	(6.0; 10.5)	8.5	(6.1; 10.8)
CCL4	Pl	8.0	(6.6; 11.1)	11.1	(8.9; 13.7)	12.5	(9.7; 17.9)	8.9	(6.0; 11.4)	9.1	(6.6; 11.3)	9.2	(6.7; 11.7)
[pg/ml] x10^{5}	Tr	9.3	(6.8; 11.8)	12.5	(8.9; 15.4)	14.1	(11.9; 16.3)	9.5	(6.6; 11.7)	9.0	(7.6; 10.9)	9.6	(6.9; 10.4)
CCL11	Pl	2.6	(1.9; 3.0)	2.5	(1.8; 2.8)	2.3	(1.7; 2.7)	2.4	(1.8; 2.9)	2.4	(1.8; 3.0)	2.6	(1.8; 2.9)
[pg/ml] x10^{2}	Tr	2.4	(1.8; 3.0)	2.2	(1.6; 2.9)	2.3	(1.7; 2.6)	2.3	(1.6; 3.1)	2.1	(1.7; 2.7)	2.2	(1.7; 3.0)
Factor VII	Pl	2.2	(1.9; 2.4)	2.1	(1.9; 2.4)	2.2	(1.9; 2.4)	2.1	(1.9; 2.3)	2.2	(2.2; 2.4)	2.3	(2.0; 2.5)
[ng/ml] x10^{2}	Tr	2.2	(1.8; 2.5)	2.1	(1.7; 2.4)	2.1	(1.8; 2.4)	2.2	(1.8; 2.4)	2.1	(1.8; 2.5)	2.2	(1.8; 2.6)
ICAM-1	Pl	46.0	(39.0; 53.0)	50.0	(43.0; 59.0)	47.0	(42.0; 54.0)	46.0	(42.0; 57.0)	46.0	(41.0; 57.0)	50.5	(43.0; 59.0)
[pg/ml]	Tr	51.5	(43.0; 59.0)	51.0	(44.0; 62.0)	50.0	(45.0; 58.0)	50.0	(44.0; 58.0)	49.0	(43.0; 58.0)	50.5	(43.0; 59.0)
IFN-γ	Pl	3.6	(0.8; 6.7)	5.8	(1.9; 12.5)	3.5	(1.4; 7.6)	3.7	(1.2; 7.6)	4.4	(1.8; 6.4)	3.8	(1.5; 8.5)
[pg/ml] x10^{3}	Tr	3.6	(2.0; 7.7)	5.9	(2.3; 13.6)	5.6	(2.3; 9.5)	5.6	(2.4; 9.2)	5.0	(1.8; 10.6)	4.4	(2.4; 8.4)
IL-1α	Pl	0.2	(0.2; 0.3)	0.4	(0.3; 0.5)	0.4	(0.3; 0.5)	0.3	(0.2; 0.4)	0.3	(0.2; 0.4)	0.3	(0.2; 0.3)
[ng/ml]	Tr	0.3	(0.1; 0.3)	0.4	(0.2; 0.5)	0.4	(0.3; 0.5)	0.3	(0.2; 0.4)	0.3	(0.2; 0.4)	0.3	(0.1; 0.4)
IL-1β	Pl	10.0	(7.4; 13.9)	13.6	(9.3; 18.7)	16.7	(13.1; 23.3)	12.9	(9.1; 17.0)	12.2	(8.7; 16.5)	11.9	(8.5; 15.6)
[pg/ml] x10^{3}	Tr	12.1	(7.9; 17.3)	14.4	(8.6; 20.2)	16.7	(14.1; 23.2)	14.3	(8.7; 18.9)	12.7	(7.5; 18.1)	12.5	(7.8; 16.3)
IL-1β serum	Pl	4.5	(3.9; 5.1)	5.2	(4.4; 6.3)	4.7	(3.8; 5.3)	4.3	(3.6; 5.2)	4.4	(3.8; 5.2)	4.5	(3.5; 5.1)
[pg/ml]	Tr	4.3	(3.7; 5.4)	5.3	(4.4; 6.4)	4.7	(3.9; 5.5)	4.2	(3.7; 5.2)	4.5	(3.5; 5.2)	4.8	(4.0; 5.4)
IL-1ra	Pl	5.7	(4.7; 6.9)	7.5	(6.2; 9.0)	10.7	(8.3; 12.8)	6.3	(5.1; 7.5)	6.3	(5.2; 7.3)	6.2	(5.0; 7.3)
[pg/ml] x10^{3}	Tr	6.4	(4.9; 8.2)	8.1	(6.6; 10.4)	11.1	(8.4; 14.1)	6.6	(5.4; 8.0)	6.6	(5.6; 8.5)	6.3	(5.2; 7.5)
IL-1ra serum	Pl	0.8	(0.6; 1.0)	1.0	(0.8; 1.1)	0.8	(0.7; 1.0)	0.7	(0.6; 1.0)	0.8	(0.6; 1.0)	0.8	(0.6; 1.0)
[pg/ml] x10^{3}	Tr	0.9	(0.7; 1.1)	1.1	(0.9; 1.3)	0.8	(0.7; 1.1)	0.8	(0.6; 1.0)	0.9	(0.7; 1.1)	0.9	(0.7; 1.1)
IL-2	Pl	0.9	(0.2; 1.9)	0.7	(0.1; 2.3)	0.6	(0.2; 2.1)	0.7	(0.2; 2.2)	0.8	(0.3; 1.9)	1.1	(0.2; 2.4)
[pg/ml] x10^{3}	Tr	1.5	(0.4; 2.6)	1.2	(0.3; 2.3)	1.6	(0.6; 3.5)	1.6	(0.6; 2.4)	1.5	(0.7; 2.6)	1.4	(0.6; 2.5)
IL-4	Pl	2.4	(1.1; 3.4)	2.0	(1.1; 3.2)	2.2	(1.1; 3.8)	2.0	(1.1; 2.8)	2.0	(1.2; 3.2)	2.2	(1.1; 3.4)
[pg/ml] x10^{2}	Tr	2.3	(1.4; 3.7)	2.2	(1.3; 3.4)	3.0	(1.7; 4.2)	2.5	(1.5; 3.7)	2.5	(1.3; 3.4)	2.6	(1.6; 3.6)
IL-5	Pl	9.8	(4.4; 18.0)	6.1	(3.4; 19.0)	12.0	(3.5; 24.0)	9.6	(3.9; 16.0)	8.9	(4.4; 17.0)	10.0	(4.1; 19.5)
[pg/ml]	Tr	13.0	(4.7; 21.0)	11.0	(4.8; 22.0)	14.5	(6.7; 25.0)	12.0	(5.9; 23.0)	12.5	(5.6; 22.0)	14.0	(4.7; 22.0)
IL-6	Pl	5.3	(4.1; 8.0)	8.1	(6.2; 11.0)	9.3	(6.5; 13.5)	6.3	(4.5; 7.6)	6.4	(4.9; 8.5)	6.0	(5.1; 8.3)
[pg/ml] x10^{4}	Tr	6.6	(4.9; 8.6)	9.0	(6.2; 12.3)	10.0	(8.2; 14.2)	7.2	(4.7; 9.1)	6.4	(4.9; 9.0)	6.1	(5.0; 8.4)
IL-6 serum	Pl	4.5	(4.5; 4.5)	4.5	(4.5; 4.5)	4.5	(4.5; 4.5)	4.5	(4.5; 4.5)	4.5	(4.5; 4.5)	4.5	(4.5; 4.5)
[pg/ml]	Tr	4.5	(4.5; 4.5)	4.5	(4.5; 4.5)	4.5	(4.5; 4.5)	4.5	(4.5; 4.5)	4.5	(4.5; 4.5)	4.5	(4.5; 4.5)
IL-7	Pl	1.7	(1.4; 1.8)	1.9	(1.8; 2.1)	1.9	(1.7; 2.1)	1.6	(1.5; 2.0)	1.7	(1.5; 1.9)	1.6	(1.5; 1.8)
[pg/ml] x10^{2}	Tr	1.7	(1.4; 1.9)	1.9	(1.8; 2.1)	1.9	(1.6; 2.1)	1.6	(1.4; 1.9)	1.7	(1.4; 1.9)	1.6	(1.4; 2.0)
IL-10	Pl	0.9	(0.4; 1.4)	1.4	(0.8; 1.8)	1.2	(0.6; 1.7)	0.9	(0.4; 1.3)	1.0	(0.5; 1.3)	0.9	(0.4; 1.3)
[pg/m l] x10^{4}	Tr	1.1	(0.7; 1.5)	1.3	(1.0; 1.9)	1.4	(0.9; 1.8)	0.9	(0.6; 1.4)	1.0	(0.7; 1.5)	1.0	(0.7; 1.5)
IL-12p40	Pl	12.0	(8.8; 17.0)	13.0	(8.3; 17.0)	17.0	(13.0; 22.0)	12.0	(8.2; 19.0)	13.0	(10.0; 17.0)	13.0	(11.0; 17.5)
[ng/ml]	Tr	14.0	(9.4; 17.0)	14.0	(10.0; 16.0)	20.0	(13.0; 24.0)	14.0	(11.0; 18.0)	14.0	(9.6; 19.0)	15.0	(11.0; 17.0)
IL-12p70	Pl	1.0	(0.4; 2.4)	0.7	(0.4; 1.5)	0.7	(0.4; 2.0)	0.8	(0.4; 2.5)	0.9	(0.4; 1.6)	0.9	(0.4; 2.3)
[pg/ml] x10^{2}	Tr	0.9	(0.4; 1.4)	0.6	(0.4; 1.1)	0.8	(0.5; 1.2)	1.1	(0.5; 1.5)	0.6	(0.4; 1.6)	0.8	(0.4; 1.6)

IL-15	Pl	1.4 (1.2; 1.6)	1.6 (1.4; 1.8)	1.7 (1.4; 1.9)	1.5 (1.3; 1.8)	1.4 (1.2; 1.8)	1.5 (1.3; 1.7)
[ng/ml]	Tr	1.5 (1.3; 1.6)	1.7 (1.3; 1.9)	1.7 (1.5; 2.0)	1.6 (1.3; 1.8)	1.5 (1.2; 1.7)	1.5 (1.2; 1.8)
IL-17	Pl	0.7 (0.3; 1.4)	0.9 (0.3; 1.6)	1.0 (0.3; 1.7)	0.7 (0.3; 1.5)	0.9 (0.3; 1.4)	0.9 (0.3; 1.6)
[pg/ml] $x10^2$	Tr	1.1 (0.6; 1.8)	1.0 (0.5; 1.7)	1.3 (0.7; 2.2)	1.1 (0.5; 1.6)	1.1 (0.6; 1.7)	1.0 (0.6; 1.7)
IL-18	Pl	3.4 (2.6; 3.8)	3.8 (3.4; 4.6)	3.9 (3.5; 4.8)	3.5 (3.0; 4.2)	3.3 (2.9; 4.1)	3.5 (3.1; 3.9)
[pg/ml] $x10^2$	Tr	3.3 (2.8; 4.0)	3.7 (3.0; 4.6)	3.9 (3.4; 4.9)	3.6 (3.1; 4.3)	3.4 (2.9; 4.0)	3.3 (2.8; 3.9)
IL-23	Pl	17.0 (13.0; 28.0)	20.0 (14.0; 25.0)	27.0 (19.0; 35.0)	17.0 (12.0; 27.0)	21.0 (13.0; 26.0)	20.0 (15.0; 27.0)
[ng/ml]	Tr	20.5 (13.0; 27.0)	22.0 (16.0; 27.0)	28.5 (22.0; 32.0)	21.0 (14.0; 27.0)	21.0 (14.0; 27.0)	19.5 (16.0; 27.0)
TNF-α	Pl	2.1 (1.5; 3.2)	2.5 (1.6; 3.8)	2.9 (2.0; 4.5)	2.2 (1.4; 3.2)	2.4 (1.5; 3.1)	2.3 (1.7; 3.3)
[pg/ml] $x10^4$	Tr	2.2 (1.5; 2.8)	2.7 (1.9; 3.6)	3.6 (2.4; 4.4)	2.9 (1.7; 3.3)	2.4 (1.7; 3.3)	2.3 (1.9; 2.8)
TNF-α serum	Pl	14.0 (14.0; 14.0)	14.0 (14.0; 15.5)	14.0 (14.0; 14.0)	14.0 (14.0; 14.0)	14.0 (14.0; 14.5)	14.0 (14.0; 14.0)
[pg/ml]	Tr	14.0 (14.0; 14.0)	14.0 (14.0; 15.0)	14.0 (14.0; 14.0)	14.0 (14.0; 14.5)	14.0 (14.0; 14.0)	14.0 (14.0; 14.0)
TNF-β	Pl	62.0 (47.0; 71.0)	66.0 (62.0; 79.0)	66.0 (54.0; 79.0)	60.0 (46.0; 72.0)	62.0 (56.0; 76.0)	67.5 (60.5; 78.0)
[pg/ml]	Tr	65.5 (54.0; 76.0)	68.0 (59.0; 86.0)	76.0 (58.0; 87.0)	66.0 (55.0; 80.0)	68.0 (54.0; 84.0)	71.0 (47.0; 79.0)
Growth factors							
BDNF	Pl	12.0 (9.9; 14.0)	16.0 (13.0; 19.0)	13.0 (10.0; 16.0)	13.0 (9.9; 15.0)	13.0 (10.0; 15.0)	13.0 (9.9; 16.5)
[ng/ml]	Tr	14.0 (11.0; 16.0)	17.0 (14.0; 22.0)	14.0 (11.0; 17.0)	14.0 (10.0; 18.0)	14.0 (10.0; 16.0)	12.0 (11.0; 16.0)
GM-CSF	Pl	1.7 (0.5; 3.4)	2.0 (0.7; 5.1)	1.7 (0.6; 3.6)	1.5 (0.6; 3.3)	1.7 (0.7; 2.7)	1.9 (0.6; 3.3)
[pg/ml] $x10^2$	Tr	2.0 (0.8; 3.4)	2.3 (0.9; 4.0)	1.8 (1.0; 3.5)	2.4 (0.9; 3.9)	1.9 (0.8; 3.9)	1.9 (0.9; 3.1)
IL-3	Pl	2.5 (2.0; 3.4)	3.2 (2.2; 4.4)	2.8 (2.1; 3.7)	2.4 (2.0; 3.5)	2.6 (2.1; 3.6)	3.0 (2.1; 3.7)
[ng/ml] $x10^{-2}$	Tr	2.7 (2.1; 3.7)	3.3 (2.5; 4.2)	3.2 (2.2; 4.2)	2.8 (2.0; 3.5)	3.1 (2.2; 4.1)	2.9 (2.2; 3.4)
SCF	Pl	0.8 (0.6; 0.9)	1.0 (0.7; 1.1)	1.0 (0.9; 1.1)	0.8 (0.7; 1.1)	0.8 (0.7; 1.0)	0.8 (0.7; 1.0)
[pg/ml] $x10^4$	Tr	0.8 (0.6; 1.0)	1.0 (0.7; 1.2)	1.0 (0.9; 1.1)	0.9 (0.7; 1.0)	0.8 (0.6; 1.0)	0.8 (0.7; 1.0)
VEGF	Pl	2.3 (1.9; 2.8)	2.8 (2.3; 3.0)	3.0 (2.6; 3.4)	2.5 (2.1; 3.2)	2.5 (2.2; 3.1)	2.4 (2.2; 3.0)
[pg/ml] $x10^2$	Tr	2.6 (2.0; 3.0)	2.7 (2.3; 3.2)	3.0 (2.7; 3.4)	2.8 (2.2; 3.1)	2.8 (1.9; 3.1)	2.6 (1.9; 2.8)
Matrix metalloproteinases							
MMP-3	Pl	6.6 (5.6; 9.1)	15.0 (12.0; 21.0)	9.0 (7.2; 12.0)	7.4 (6.6; 10.0)	8.1 (6.4; 11.0)	7.5 (6.3; 11.0)
[ng/ml]	Tr	8.5 (5.7; 10.0)	18.0 (13.0; 23.0)	9.3 (7.7; 14.0)	8.3 (6.9; 10.0)	8.6 (7.0; 10.0)	8.3 (6.6; 12.0)
MMP-9	Pl	1.4 (1.2; 1.6)	1.6 (1.4; 1.8)	1.9 (1.7; 2.2)	1.5 (1.3; 1.7)	1.4 (1.3; 1.6)	1.4 (1.2; 1.6)
[ng/ml] $x10^2$	Tr	1.4 (1.2; 1.6)	1.6 (1.4; 1.8)	1.9 (1.6; 2.1)	1.5 (1.2; 1.7)	1.4 (1.2; 1.6)	1.5 (1.2; 1.6)
Acute phase protein							
CRP	Pl	0.4 (0.2; 0.8)	0.4 (0.2; 0.8)	0.4 (0.2; 0.8)	1.5 (0.8; 3.3)	1.0 (0.6; 2.2)	0.8 (0.4; 1.8)
[mg/L]	Tr	0.4 (0.3; 0.7)	0.4 (0.3; 0.7)	0.4 (0.3; 0.7)	1.6 (1.1; 2.5)	1.1 (0.7; 2.0)	0.8 (0.4; 1.2)

BDNF = Brain-Derived Neurotrophic Factor; CK = creatine kinase; CRP = C-reactive Protein; GM-CSF = Granulocyte Macrophage Colony-Stimulating Factor; ICAM-1 = Inter-Cellular Adhesion Molecule 1; IFN-γ = Interferon-gamma; LDH = lactate dehydrogenase; MMP = Matrix Metalloproteinase; SCF = Stem Cell Factor; TNF = Tumor Necrosis Factor; VEGF = Vascular Endothelial Growth Factor

Values: Median (1. Quartile; 3. Quartile)

Supplemental table S2:

group	Low responders [<500 U/L]	Medium responders [500-2000 U/L]	High responders [>2000 U/L]	Total	p Value
**Placebo**	n= 13<br>27.66 %	n= 32<br>68.09 %	n= 2<br>4.26 %	n= 47	0.83
**Tr14**	n= 15<br>31.25 %	n= 30<br>62.50 %	n= 3<br>6.25 %	n= 48	
**Total**	n= 28	n= 62	n= 5	n= 95	
